# Gallstone among Patients Presenting to the Department of Surgery in a Tertiary Care Center: A Descriptive Cross-sectional Study

**DOI:** 10.31729/jnma.8123

**Published:** 2023-04-30

**Authors:** Soumya Pahari, Sunil Basukala, Utsav Piya, Yugant Khand, Baivavi Thapa, Ojas Thapa, Suman Thapa

**Affiliations:** 1 Nepalese Army Institute of Health Sciences, Sanobharyang, Kathmandu, Nepal; 2 Department of Surgery, Shree Birendra Hospital, Chhauni, Kathmandu, Nepal

**Keywords:** *cholelithiasis*, *gallbladder*, *prevalence*

## Abstract

**Introduction:**

Gallstone is the most common biliary pathology. Once thought of as a disease of the western world, the incidence and burden of cholelithiasis are increasing in Asia. Its literature from Nepal is however still primitive. The study aimed to find out the prevalence of gallstone among patients presenting to the Department of Surgery in a tertiary care centre.

**Methods:**

A descriptive cross-sectional study was conducted among patients presenting to the Department of Surgery after receiving ethical approval from the Institutional Review Committee (Registration number: 625). The study was conducted from 1 June 2022 to 1 November 2022. Patients with age more than 18 years were included and patients less than 18 years of age, having common bile duct stones, suffering from biliary malignancy or having an immunocompromised state were excluded. Convenience sampling was done. Point estimate and 95% Confidence Interval were calculated.

**Results:**

Among 1700 patients, gallstone was seen in 200 (11.76%) (10.23-13.29, 95% Confidence Interval). Among the 200 patients, 133 of them (66.50%) were females. Multiple gallstones were present in 118 (59%) cases whereas 82 (41%) cases had a single stone.

**Conclusions:**

The prevalence of gallstone was found to be similar as compared to other reported literature.

## INTRODUCTION

Gallstone is the most common biliary pathology with a global incidence of 3-21.9%.^1^ In Asia, it is 4-15%.^2,3^ Most of the patients with gallstone are asymptomatic and are diagnosed incidentally.^3^ Symptomatic patients classically present with biliary colic, usually accompanied by nausea, vomiting and diaphoresis.^4^ Laparoscopic cholecystectomy is the gold standard treatment. However, about 5.2% of cases convert to open cholecystectomy for better outcomes due to various reasons.^5^

Once considered a disease of the western world, the incidence of gallstone in Asia is considerable and rising.^5-7^ Literature related to gallstone in Nepal is primitive and more studies help to understand this disease in our setting.

The study aimed to find out the prevalence of gallstone among patients presenting to the Department of Surgery in a tertiary care centre.

## METHODS

A descriptive cross-sectional study was conducted among patients presenting to the Department of Surgery of a tertiary hospital from 1 June 2022 to 1 November 2022. Ethical approval was taken from the Institutional Review Committee (IRC) of the Nepalese Army Institute of Health Sciences (NAIHS) (Registration number: 625) before the conduct of the study. All the adult patients with gallstone above the age >18 years who presented to the Department of Surgery via emergency or outpatient setting and gave informed consent were included in the study. Patients of less than 18 years of age, with common bile duct stones or suffering from biliary malignancy or having an immunocompromised state were excluded from the study. Patients whose abdominal imaging (ultrasonography or computerised tomography) demonstrated the presence of gallstones were taken as cases of gallstone. The study participants were explained about the study in detail. They were assured of confidentiality and informed written consent was taken. A convenience sampling method was adopted. The sample size was calculated by using the following formula:


$$\begin{array}{ll} \text{n} & ={\text{Z}}^{2}\times \frac{\text{p}\times \text{q}}{{\text{e}}^{\text{2}}}\\ & =1.96^{2}\times \frac{0.50\times 0.50}{0.03^{2}}\\ & =1068 \end{array}$$

Where,

n = minimum required sample size
Z = 1.96 at 95% Confidence interval (CI)
p = prevalence taken as 50% for maximum sample size calculation
q = 1-p
e = margin of error, 3%

The minimum sample size calculated was 1068. Adding 10% non response rate, sample size was 1186. However, 1700 cases were taken for the study.

Data were collected in the pre-formed proforma and entered in Microsoft Excel Version 2010 and analyzed using IBM SPSS Statistics version 24.0. Point estimate and 95% CI were calculated.

## RESULTS

**Figure 1:**
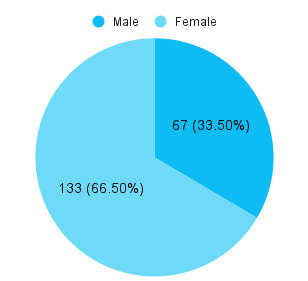
Gender-wise distribution (n= 200)

Among 1700 patients, the prevalence of gallstone was seen in 200 (11.76%) (10.23-13.29, 95%, CI). Among them, the mean age was 50.67±13.76 years. In our study, 133 (66.50%) were females and 67 (33.50%) of patients were males with a sex ratio of male:female =1:1.98 (Figure 1).

**Figure 2:**
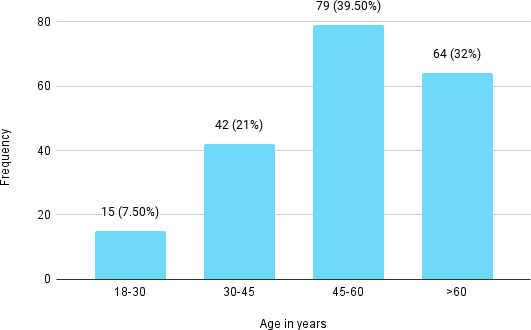
Age distribution (n= 200).

Gallstone was highest among the patients in the age group between 45-60 years, 79 (39.50%) patients and the least in the age group 18-30 years 15 (7.50%) patients (Figure 2).

Among the 200 cases, 189 (94.50%) were nonvegetarians and 11 (5.50%) were vegetarians. 17 (8.50%) cases were smokers. Obesity (BMI ≥30 kg/m^2^) was present in 38 (19%) cases and 64 (32%) cases were overweight (BMI 25-29.9 kg/m^2^) (Table 1).

**Table 1:** Underlying conditions (n = 200).

Variables		n (%)
Diet	Non-vegetarian	189 (94.50)
	Vegetarian	11 (5.5)
Smoking Habits	Smoker	17 (34)
BMI	Obese	38 (19)
	Overweight	64 (32)
	Underweight	-

Majority of patients complained of abdominal pain 196 (98%) followed by Vomiting 60 (30%) (Table 2).

**Table 2:** Clinical findings (n= 200).

Variables	n (%)
Abdominal Pain	196 (98)
Vomiting	60 (30)
Dyspepsia	12 (6)
Jaundice	20 (10)
Palpable gallbladder	9 (4.50)
Murphy's Sign	22 (11)

On Ultrasonography (USG) single stone and multiple stones was seen in 82 (41%) and 118 (59%) cases respectively. Laboratory and USG findings are summarized in (Table 3).

**Table 3:** Laboratory and ultrasonography findings in gallstone.

Investigation findings	Mean±SD
Leukocytosis (cells/pl)	8216.49±3660.71
Total bilirubin (mg/dl)	0.680±0.665

Among the total patients, 40 (20%) were managed conservatively, 154 (77%) underwent laparoscopic cholecystectomy and 6 (3%) underwent open cholecystectomy (Table 4).

**Table 4:** Mode of management of gallstone (n = 200).

Mode of management	n (%)
Conservative management	40 (20)
Laparoscopic cholecystectomy	154 (77)
Open cholecystectomy	6 (3)

## DISCUSSION

Gallstone is a common gastrointestinal condition worldwide that remains asymptomatic in the majority of patients. This highly prevalent condition occurs in almost 15-20% of the western world and affects 10% of the population in Asian countries like Japan.^1,3^ Similar prevalence rate of 11.76% was found in our study. Most studies conclude that in all age groups of patients with gallstones, females are more affected than males.^2-4^ Consistent with other studies, we report that 66.5% of patients were female while 33.5% were male. Gender has a powerful influence on gallstones and females are twice as likely as males to acquire gallstones. Pregnancy, the use of oral contraceptive pills and the effect of female sex hormones on gallstone formation are well-known contributing factors.^8^ In a study done in Nepal, cholelithiasis was common in the age group of 31-40 years.^4^ However, our study showed a higher prevalence at 45-60 years of age.

Among the 200 cases, 94.50% were non-vegetarians and 5.5% were vegetarians. A similar study done in Nepal showed 92.7% of patients were non-vegetarians which is similar to the findings of our study.^9^ A similar study done in India showed that 58% of cases consumed a non-vegetarian diet which is less than the findings of our study.^10^ Studies have shown a positive association between cholesterol gallstones and consumption of a high fat-containing diet and red meat.^11^ 34% of cases were smokers and obesity (BMI ≥ 30 km/m2 ) was present in 19% of cases. The prevalence of obesity in gallstone patients was 19%. This finding is lower compared to a similar study where obesity was present in 26.24%.^9^ The reason for this may be that this hospital receives military patients who may be more fit than the general population. Obesity is a well-established risk factor for gallstone and at least 25% of morbidly obese individuals have gallstones. The underlying mechanism is obesity leads to increased hepatic cholesterol synthesis and increased secretion of cholesterol in the bile.^3^

In our study, 10.5% of cases were asymptomatic and detected incidentally. A previous study done in Nepal showed 8.42% were asymptomatic, similar to our findings.^9^ However, another study done in India showed a higher percentage of asymptomatic cases 15.5% compared to our study.^1^ The reason for the lesser number of patients being detected at the asymptomatic stage may be due to the poor health-seeking behaviour of the population in our setting. Most of the patients with gallstones remain asymptomatic and the rate of complication of incidental cholelithiasis per year is almost half of that compared to symptomatic cholelithiasis.^12^ Almost all the patients 98%, had abdominal pain.

Nausea or vomiting was present in 30% of cases. Previous studies have reported nausea and vomiting among 64.4%^10^ and 56%^13^ of patients respectively had nausea or vomiting, which is more compared to our study. Biliary colic is a typical presentation of gallstone characterized by intermittent episodes of constant, sharp, right upper quadrant abdominal pain often associated with nausea and vomiting.^14^ The prevalence of jaundice in gallstone patients reported by similar studies is variable, ranging from 1.08% to 16.5%.^1,9^ In our study, 10% of cases had jaundice. Jaundice is often a feature of choledocholithiasis when the gallstone gets dislodged and impacted into the common bile duct leading to obstruction. Murphy's sign (tenderness over the gallbladder) was present in 11% of cases and the gallbladder was palpable in 4.5% cases. A previous study reported that Murphy's sign was positive in only 4.86% of cases whereas the gallbladder was palpable in only 1.08% cases.^12^ Murphy's sign usually indicates acute cholecystitis, with a sensitivity ranging from 58-71%.^15^ Courvoirsier's law states that a palpable gallbladder is unlikely due to gallstones, however Courvoisier observed that 20% of patients with obstruction of common bile duct with gallstones had a palpable gallbladder.^16^

Leukocytosis (total leucocytes count more than 11,000/ mm^3^ ) was present in 22% of cases. Hyperbilirubinemia (Total bilirubin >1.2 mg/dl) was present in 13% patients. Underlying complications of gallstone may be responsible for unusual clinical features like fever, palpable gallbladder, jaundice and leukocytosis. In ultrasonography of the abdomen, 59% patients had multiple stones and 41% patients had a single stone in the gallbladder. Previous studies have reported multiple gallstones present in 76%^13^ and 73.7%^6^ of patients with gallstone, which is more compared to our study. Multiple small gallstones are a risk factor for synchronous asymptomatic common bile duct stones.^8^

In our study, the majority of the patients were treated by laparoscopic cholecystectomy 77%. About 20% cases were treated conservatively with follow-up for reasons like asymptomatic disease and unfit for surgery. Only 3% cases were treated by open cholecystectomy. A similar study reported that 1.9% of cases of gallstone underwent open cholecystectomy.^9^ Previous studies showed 53.8%^10^ and 50%^13^ cases underwent laparoscopic cholecystectomy respectively which is less compared to our findings. Numerous treatments of cholelithiasis have been described. Asymptomatic cases can be treated conservatively with annual follow-up. In select populations, oral bile acid dissolution therapy and extracorporeal shock wave lithotripsy (ESWL) have also been practised successfully. Laparoscopic cholecystectomy is considered the first-line surgical therapy for symptomatic gallstones due to less complications and faster recovery time. However, if concomitant gallbladder cancer is suspected preoperatively, open cholecystectomy is the procedure of choice. Laparoscopic cholecystectomy may be converted to open if situations like difficult anatomy arise intraoperatively.^17^

Our study did not include description of imaging findings, histopathology, composition of gallstones and intraoperative/ postoperative complications. Also, the findings of this single institution study may not be generalizable to the entire population.

## CONCLUSIONS

The prevalence of gallstone was found be similar as compared to other reported literature. Multicenter prospective studies are recommended to better understand the clinical-epidemiological features and outcomes of this common surgical disease in Nepal.
